# Long‐term market value after meniscal injury in professional football players largely follows expected career trajectories: A matched cohort study

**DOI:** 10.1002/jeo2.70968

**Published:** 2026-10-29

**Authors:** Riccardo D'Ambrosi, Jacopo Conteduca, Andrea Mei, Marco Sorrentino, Claudio Cordani, Christian Fink, Philipp Baumert

**Affiliations:** ^1^ IRCCS Ospedale Galeazzi – Sant'Ambrogio Milan Italy; ^2^ Link Campus University Rome Italy; ^3^ Ospedale Vito Fazzi Lecce Italy; ^4^ Residency Program in Orthopaedic and Traumatology University of Milan Milan Italy; ^5^ 10X Educational Division Milan Italy; ^6^ Research Unit for Orthopaedic Sports Medicine and Injury Prevention, Institute for Sports Medicine, Alpine Medicine and Health Tourism UMIT TIROL‐Private University for Health Sciences and Health Technology Hall Tirol Austria; ^7^ Gelenkpunkt‐Sports and Joint Surgery, FIFA Medical Centre of Excellence Innsbruck/Austria Olympiastraße Innsbruck Austria; ^8^ School of Sport and Exercise Sciences Liverpool John Moores University Liverpool UK

**Keywords:** economic impact, longitudinal analysis, market value, matched cohort, meniscal injury, professional football, soccer

## Abstract

**Purpose:**

To evaluate long‐term changes in market value following meniscal injury in professional football players compared with matched players without meniscal injury and identify characteristics associated with postinjury market value.

**Methods:**

This retrospective cohort study analysed 80 meniscal injury episodes (74 players) recorded in Transfermarkt between 2006 and 2024. Market value was assessed during the preinjury season, injury year and annually for five subsequent years. Linear mixed‐effects models evaluated longitudinal changes and associations with age, playing position and competitive level. Injury episodes identifiable in an openly available Transfermarkt‐derived database were matched (1:2) to controls without recorded meniscal injury by position, competitive level, calendar year, age and preinjury market value. Covariate balance was assessed using standardised mean differences (SMDs), and trajectories were compared between groups.

**Results:**

Mean age at injury was 25.05 ± 4.36 years, and mean absence was 103.87 ± 93.48 days. Market value increased at one (+25.61%; *p* = 0.023) and 2 years (+26.82%; *p* = 0.019) compared with preinjury values, before declining by 31.56% at 5 years (*p* = 0.001). Older age and lower competitive level were independently associated with lower market value. Among 64 matched injury episodes and 128 controls (all SMDs <0.15), market value declined in both groups over 5 years (−44.8% vs. −34.5%), without significant between‐group differences at any time point (5‐year difference, −15.7%; 95% CI, −39.2% to +16.8%; *p* = 0.30). Sensitivity analyses showed no significant additional decline among injured players.

**Conclusion:**

Meniscal injuries were not associated with an immediate reduction in market value among professional football players. The long‐term decline in market value did not differ significantly from that of matched players without meniscal injury and appeared largely to reflect the expected age‐related evolution of player value; however, given the width of the confidence intervals and the absence of clinical detail, a moderate injury‐related effect cannot be excluded.

**Level of Evidence:**

Level III, retrospective cohort study.

## Graphical abstract

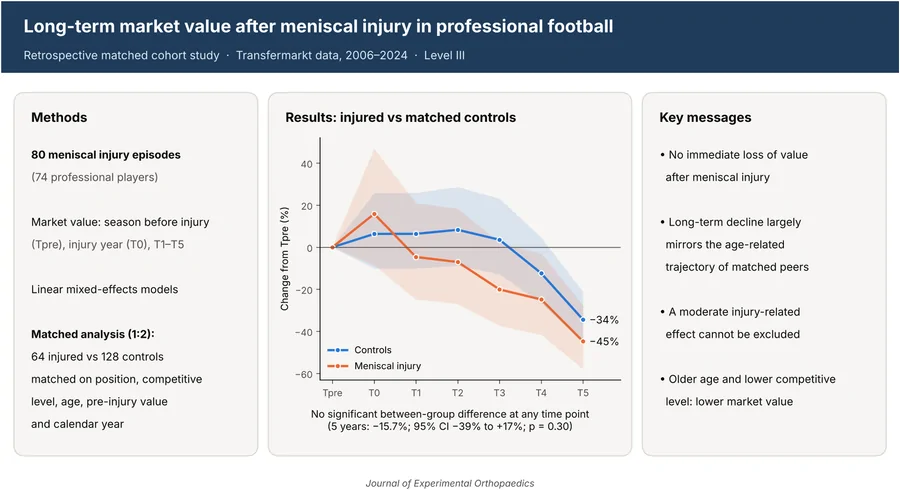

**Abbreviations**
ANOVA: analysis of variance
CI: confidence interval
ICC: intraclass correlation coefficient
LMM: linear mixed‐effects model
REML: restricted maximum likelihood
SD: standard deviation
SMD: standardised mean difference

## INTRODUCTION

Meniscal injuries are among the most common knee injuries in professional football and represent a substantial cause of time loss [16]. In elite cohorts, they are associated with prolonged unavailability, and the layoff they impose is among the longer ones for common football injuries [8]. In the present cohort, the mean injury‐related absence exceeded 3 months, underscoring the burden that meniscal injuries place on both players and clubs.

The management of meniscal injuries ranges from meniscectomy to meniscal repair, and although most professional athletes return to sport, functional recovery is not always complete [2]. Return to sport after meniscal repair requires a longer and lesion‐specific rehabilitation [3], and failure rates after repair differ according to tear pattern and compartment [7]. Partial meniscectomy in particular has been associated with an increased long‐term risk of knee osteoarthritis [17], and even minor concomitant meniscal injuries have been associated with posttraumatic osteoarthritis 15 years after anterior cruciate ligament reconstruction [13], while among elite soccer players meniscal tears have been shown to reduce subsequent performance despite high rates of return to play [14]. These observations suggest that the consequences of meniscal injury may extend well beyond the return‐to‐play milestone.

In professional football, a player's market value is widely used as an integrated proxy of expected performance, age and potential, and publicly available databases such as Transfermarkt have enabled the longitudinal study of how injuries relate to players' economic value and careers [5, 6]. For anterior cruciate ligament injuries, such analyses have documented measurable reductions in performance, career length and economic value [12]. A significant decline in market value has been reported after proximal hamstring repair despite reliable return to play [4], and predicted injury risk itself has been shown to depress transfer values [18]. Whether meniscal injuries carry a comparable long‐term economic signature, however, remains unknown.

Despite the frequency of meniscal injuries and their potential functional and degenerative sequelae, the long‐term trajectory of players' market value following meniscal injury remains unclear. Importantly, longitudinal changes observed after injury cannot be assumed to represent an injury‐specific effect, because market value is strongly influenced by age and the natural evolution of a professional football career. The purpose of this study was therefore to evaluate long‐term changes in market value following meniscal injury in professional football players and to determine whether these changes differed from those observed in matched players without meniscal injury. A secondary aim was to identify player characteristics associated with postinjury market value. It was hypothesised that market value would decline over long‐term follow‐up after meniscal injury, but that the magnitude of this decline would not substantially exceed that observed in comparable players without meniscal injury.

## MATERIALS AND METHODS

### Study design and data source

This retrospective cohort study analysed publicly available data obtained from Transfermarkt, an online database providing information on professional football players, including injury history, player characteristics, competition level and estimated market value. The study investigated longitudinal changes in market value following documented meniscal injuries in professional football players.

The database included injuries sustained between July 2006 and January 2024 by players competing in European professional football leagues. Recorded competitions included the German Bundesliga and 2. Bundesliga, Spanish LaLiga and LaLiga2, Italian Serie A and Serie B, English Premier League and Championship, French Ligue 1, and other European leagues and lower‐tier competitions. Injury episodes were obtained from a previously compiled extraction of Transfermarkt injury records (data export of March 2025), prepared by one of the authors, which included 106 records coded as meniscal injury in players competing in European professional leagues. Market values, injury‐related absence and follow‐up information were subsequently completed for each record by one author using the player profiles on transfermarkt.com (Figure 1).

**Figure 1:**
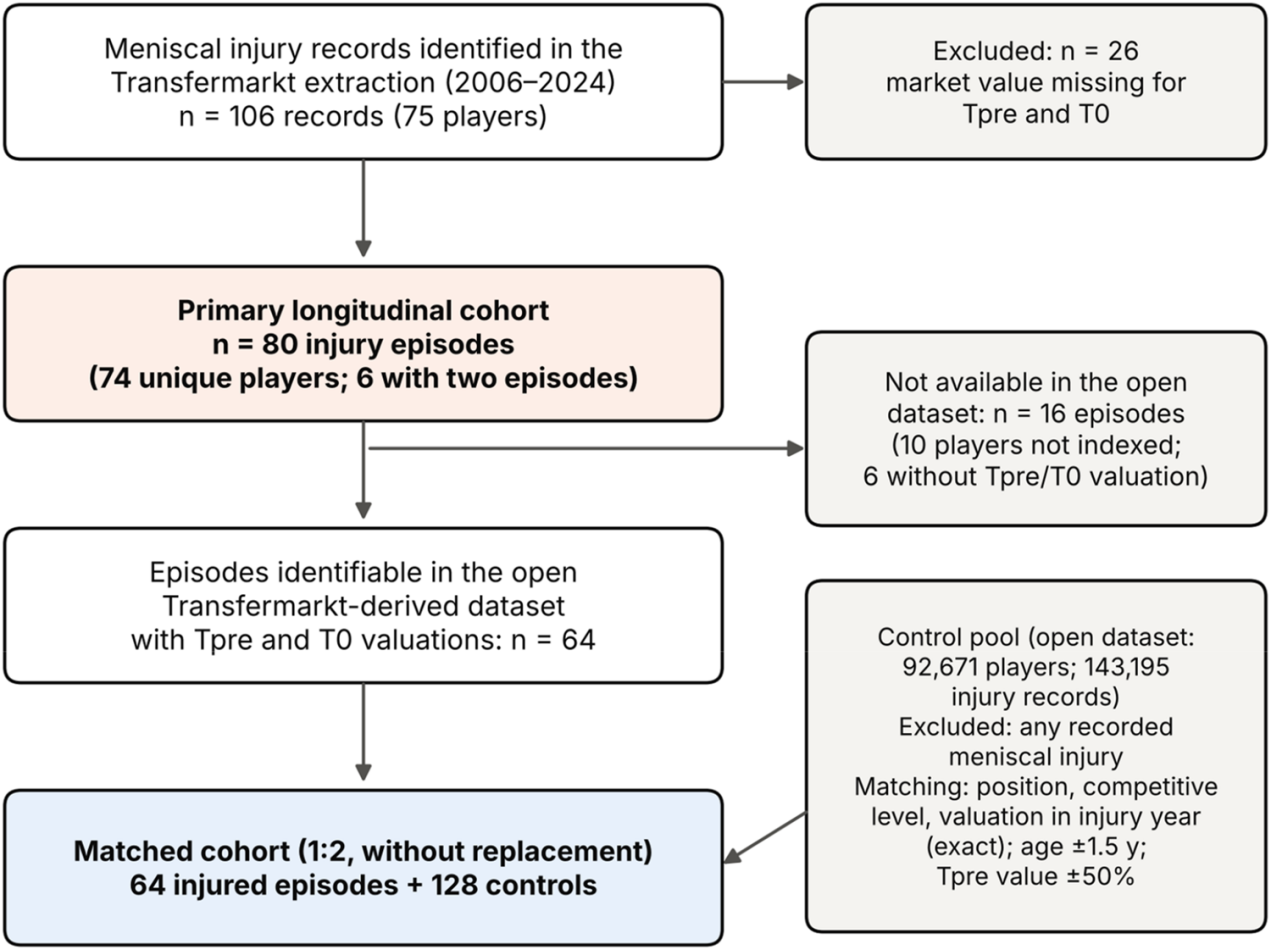
Flow diagram of injury episode selection for the primary longitudinal cohort and the matched analysis.

For each injury episode, information was collected regarding player demographics, playing position, preferred foot, height, league, injury dates, injury‐related absence and estimated market value before and after the injury. Market values were extracted from Transfermarkt and recorded for the season preceding the injury, the injury period and up to five subsequent annual time points.

Because Transfermarkt is a publicly available, community‐driven database, the accuracy and completeness of injury‐related information depend on publicly reported data. Consequently, detailed clinical information, including meniscal tear morphology, affected compartment, concomitant injuries, treatment strategy and surgical technique, was not consistently available [11].

#### Injury Definition and Inclusion Criteria

Injury episodes were included if a meniscal injury was documented in the Transfermarkt database and both injury onset and recovery dates were available. Eligible records also required information on playing position and available market value assessments for both the season preceding the injury (Tpre) and the injury year (T0).

All playing positions were considered, including goalkeepers, defenders, midfielders and forwards. Meniscal injuries were identified using the injury classification codes recorded in the database. Five Transfermarkt injury categories (original German labels with numeric identifiers) were included: ‘Meniskusverletzung’ (meniscal injury; code 211), ‘Meniskusschaden’ (meniscal damage; code 7), ‘Meniskuseinriss’ (meniscal tear; code 150), ‘Meniskusriss’ (meniscal rupture; code 348) and ‘Außenmeniskuseinriss’ (lateral meniscal tear; code 151). Because these labels are assigned by the database rather than derived from clinical records and do not consistently specify the affected compartment, lesion type or treatment, all categories were analysed together; their distribution is reported in Supporting Information: Table S2.

Injury‐related absence was defined as the number of days between the recorded injury onset and the end of the injury period. This measure was interpreted as the duration of documented unavailability rather than a confirmed return to official match participation, because match‐level return‐to‐play data were not available in the dataset.

When a player sustained more than one eligible meniscal injury, each injury was treated as a separate and independent injury episode. Accordingly, the injury episode, rather than the individual player, was considered the unit of analysis. Repeated market value measurements obtained before and after each injury were accounted for using an injury‐episode‐specific random intercept. Because six players contributed two episodes each, the primary longitudinal model was repeated as a sensitivity analysis restricted to the first recorded episode of each player.

#### Variables and outcomes

The primary outcome was the longitudinal change in estimated market value following meniscal injury.

Market value was assessed at seven predefined time points: the season preceding the injury (Tpre), the injury period (T0) and the first through fifth subsequent annual assessments (T1–T5). Values originally reported in thousands or millions of euros were converted into a consistent numerical format before analysis.

The primary exposure variable was time relative to injury, assessed both categorically across individual observation points and continuously in years relative to the injury period. Additional variables included age at injury, playing position, league or league category, preferred foot, height, injury‐related absence and the season during which the injury occurred.

Age at injury was calculated from the recorded date of birth and injury onset or obtained from the corresponding database field. Playing position was categorised as goalkeeper, defender, midfielder or forward. The timing of the injury within the calendar year was classified as winter, spring, summer or autumn.

Market value assessments recorded as ‘retired’ were not treated as numerical values. These observations were considered unavailable for subsequent market value analyses, and their implications for potential attrition bias were acknowledged.

#### Matched control‐group analysis

To determine whether longitudinal changes in market value following meniscal injury exceeded those expected from the natural evolution of player value over time, injured players were compared with matched controls. Market values for both injured players and controls were obtained from an openly available, Transfermarkt‐derived dataset comprising 92,671 player profiles, 901,457 historical market‐value records, and 143,195 injury histories, ensuring that market value was measured using an identical method in both groups. This dataset was used only to source control players and to re‐measure market values in both groups; injured players were not identified from it. Of the 80 eligible injury episodes included in the longitudinal cohort, 64 could be identified in this dataset and had retrievable preinjury (Tpre) and injury‐year (T0) market values and were therefore included in the matched analysis [14] (Figure 1).

In this analysis, market value was defined, for both groups, as the most recent valuation recorded on or before each reference date: 1 year before the injury date (Tpre), the injury date (T0) and each of the five subsequent anniversaries (T1–T5). Valuations recorded more than 2 years before a reference date were treated as missing to avoid carrying forward values of players who were no longer active.

Each injury episode was matched to two control players with no recorded meniscal injury at any time in the dataset. Matching was performed using exact matching for playing position, competitive level (first tier, second tier or third tier or lower, defined as the league in which the player recorded the most minutes during the season of injury; for injured players without league data in the open dataset, the competitive level recorded in the primary dataset was used), and active market valuation during the corresponding injury year, together with caliper matching for age (within 1.5 years) and preinjury market value (within 50%). Controls were randomly selected within each matched stratum using a fixed random seed (seed = 42) and without replacement, yielding 128 matched controls [14].

Covariate balance after matching was assessed with SMDs for age, preinjury market value (log‐transformed), calendar year, height, playing position, competitive level and preferred foot; for categorical variables, the multivariate SMD was calculated. An absolute SMD < 0.10 was considered negligible and <0.20 small [1].

### Statistical analysis

Descriptive statistics were reported as absolute frequencies and percentages for categorical variables and as means with standard deviations (SDs) and medians with ranges for continuous variables. Correlations between variables were assessed using Pearson correlation coefficients.

To evaluate changes in football players' market value over time, means, SDs, medians and ranges were calculated for each time point. Differences between time points were subsequently assessed using a linear mixed‐effects model (LMM). The natural logarithm of market value was included as the dependent variable, whereas the time point was entered as a categorical fixed effect. An injury‐episode‐specific random intercept was incorporated to account for the nonindependence of repeated measurements within each injury episode. The year preceding the injury (Tpre) was used as the reference category to evaluate differences relative to preinjury market value. As a sensitivity analysis, the model was repeated using the year of injury (T0) as the reference category [10].

To assess differences in market value according to playing position, the same modelling approach was applied, with playing position included as a categorical fixed effect instead of time point. Differences according to playing position were also assessed separately at each time point using one‐way analysis of variance (ANOVA), followed by Dunnett's post hoc test [15].

To further investigate changes in market value while adjusting for potential confounders, a multivariable LMM was fitted, with the natural logarithm of market value as the dependent variable and an injury‐specific random intercept. Fixed effects included time, expressed continuously as years relative to injury; a binary indicator of the postinjury period, defined as time points after T0; the interaction between time and postinjury status; age at injury; playing position and league category. Model‐based predicted market values, expressed in thousands of euros (€1000), were also estimated at the median age for each playing position and league category [10].

All LMMs were fitted using restricted maximum likelihood (REML). Model coefficients were expressed as percentage changes using the transformation [(exp[β] − 1) × 100], with corresponding 95% confidence intervals calculated using the Wald method. The intraclass correlation coefficient (ICC) was calculated for each LMM to quantify the proportion of total variance attributable to differences between injury episodes. The normality of variables and model residuals was assessed using the Shapiro–Wilk test [9].

Differences between injured players and controls were evaluated with a LMM including time point, group and their interaction, with a player‐specific random intercept; the group‐by‐time‐point interaction quantified the difference in market value change from baseline between injured players and matched controls.

Sensitivity analyses comprised (i) restriction of the primary longitudinal model to the first episode of each player; (ii) comparison of trajectories between tear‐specific injury labels (codes 150, 151 and 348) and nonspecific labels (codes 7 and 211); (iii) comparison of trajectories between episodes with injury‐related absence above and below the median, used as a surrogate of injury severity because tear location and treatment were not available; (iv) repetition of the matched analysis without matching on competitive level; (v) restriction of the control pool to players with no recorded lower‐extremity injury of any type and (vi) repetition of the random control selection with 100 alternative seeds. Sensitivity analyses (ii) and (iii) were evaluated with time‐point‐by‐subgroup interaction terms.

All statistical tests were two‐sided, and a *p*‐value < 0.05 was considered statistically significant. Statistical analyses were performed using R software, version 4.1.1 (R Foundation for Statistical Computing); the matched and sensitivity analyses were performed in Python 3.13 with the statsmodels package (version 0.15).

## RESULTS

### Baseline characteristics

A total of 80 meniscal injury episodes in professional football players were included in the analysis (Table 1, Figure 1). These episodes involved 74 unique players: 68 players contributed one episode, and 6 players contributed two episodes. Of the 106 meniscal injury records initially identified, 26 were excluded because market values were unavailable for both Tpre and T0. Nonspecific meniscal injury (code 211; *n* = 31, 38.8%) and meniscal damage (code 7; *n* = 19, 23.8%) were the most frequent categories, followed by meniscal tear (code 150; *n* = 14, 17.5%), meniscal rupture (code 348; *n* = 11, 13.8%), and lateral meniscal tear (code 151; *n* = 5, 6.2%); no category specified the medial meniscus or the treatment performed (Supporting Information: Table S2).

**Table 1:** Baseline characteristics and injury profile of professional football players with meniscal injuries.

	Overall (*N* = 80), *n* (%)
Role	
Defender	36 (45.0)
Forward	11 (13.8)
Goalkeeper	8 (10.0)
Midfielder	25 (31.2)
Foot	
Both	10 (12.5)
Left	16 (20.0)
Right	54 (67.5)
Height (m), mean ± SD	1.83 ± 0.06
League	
Bundesliga	22 (27.5)
2. Bundesliga	14 (17.5)
LaLiga	10 (12.5)
LaLiga2	9 (11.2)
3. Liga	2 (2.5)
Serie A	7 (8.8)
Serie B	1 (1.2)
Serie C	2 (2.5)
Championship	3 (3.8)
Eredivisie	2 (2.5)
Ligue 1	2 (2.5)
Premier League	2 (2.5)
Prva Liga	1 (1.2)
Regionalliga Northeast	2 (2.5)
Super League	1 (1.2)
Competitive level^a^	
Level 1	47 (58.8)
Level 2	27 (33.7)
Level 3	6 (7.5)
Age at injury (years), mean ± SD	25.05 ± 4.36
Season	
Spring	16 (20.0)
Summer	17 (21.2)
Autumn	26 (32.5)
Winter	21 (26.2)
Year of injury	
2006	1 (1.2)
2007	2 (2.5)
2008	4 (5.0)
2009	8 (10.0)
2010	5 (6.2)
2011	6 (7.5)
2012	2 (2.5)
2013	2 (2.5)
2014	3 (3.8)
2015	4 (5.0)
2016	6 (7.5)
2017	11 (13.8)
2018	3 (3.8)
2019	9 (11.2)
2020	2 (2.5)
2021	7 (8.8)
2022	1 (1.2)
2023	2 (2.5)
2024	2 (2.5)
Injury‐related absence (days),^b^ mean ± SD	103.87 ± 93.48
Unique players/players with two episodes	74/6

Abbreviation: SD, standard deviation.

^a^ Level 1 included Serie A, LaLiga, Bundesliga, Eredivisie, Ligue 1, Super League, Prva Liga, and the Premier League. Level 2 included Serie B, 2. Bundesliga, the Championship, and LaLiga2. Level 3 included Serie C, 3. Liga, and Regionalliga Northeast.

^b^ Injury‐related absence had one missing value.

The mean age at injury was 25.05 ± 4.36 years, and the mean height was 1.83 ± 0.06 m. Defenders represented the largest positional group (*n* = 36; 45.0%), followed by midfielders (*n* = 25; 31.2%), forwards (*n* = 11; 13.8%) and goalkeepers (*n* = 8; 10.0%). Most players were right‐footed (*n* = 54; 67.5%), whereas 16 (20.0%) were left‐footed and 10 (12.5%) were two‐footed.

The most represented competitions were the Bundesliga (*n* = 22; 27.5%), 2. Bundesliga (*n* = 14; 17.5%), LaLiga (*n* = 10; 12.5%), LaLiga2 (*n* = 9; 11.2%) and Serie A (*n* = 7; 8.8%). Overall, 47 injury episodes (58.8%) involved players competing in first‐tier leagues, 27 (33.7%) involved players in second‐tier leagues and 6 (7.5%) involved players in third‐tier competitions.

Injuries occurred between 2006 and 2024, with the highest frequencies observed in 2017 (*n* = 11; 13.8%), 2019 (*n* = 9; 11.2%) and 2009 (*n* = 8; 10.0%). Regarding seasonal distribution, 26 injuries (32.5%) occurred in autumn, 21 (26.2%) in winter, 17 (21.2%) in summer and 16 (20.0%) in spring. The mean duration of injury‐related absence was 103.87 ± 93.48 days, with one missing observation.

### Correlation analysis

Significant correlations were observed between market values across all assessment time points (Figure 2).

**Figure 2:**
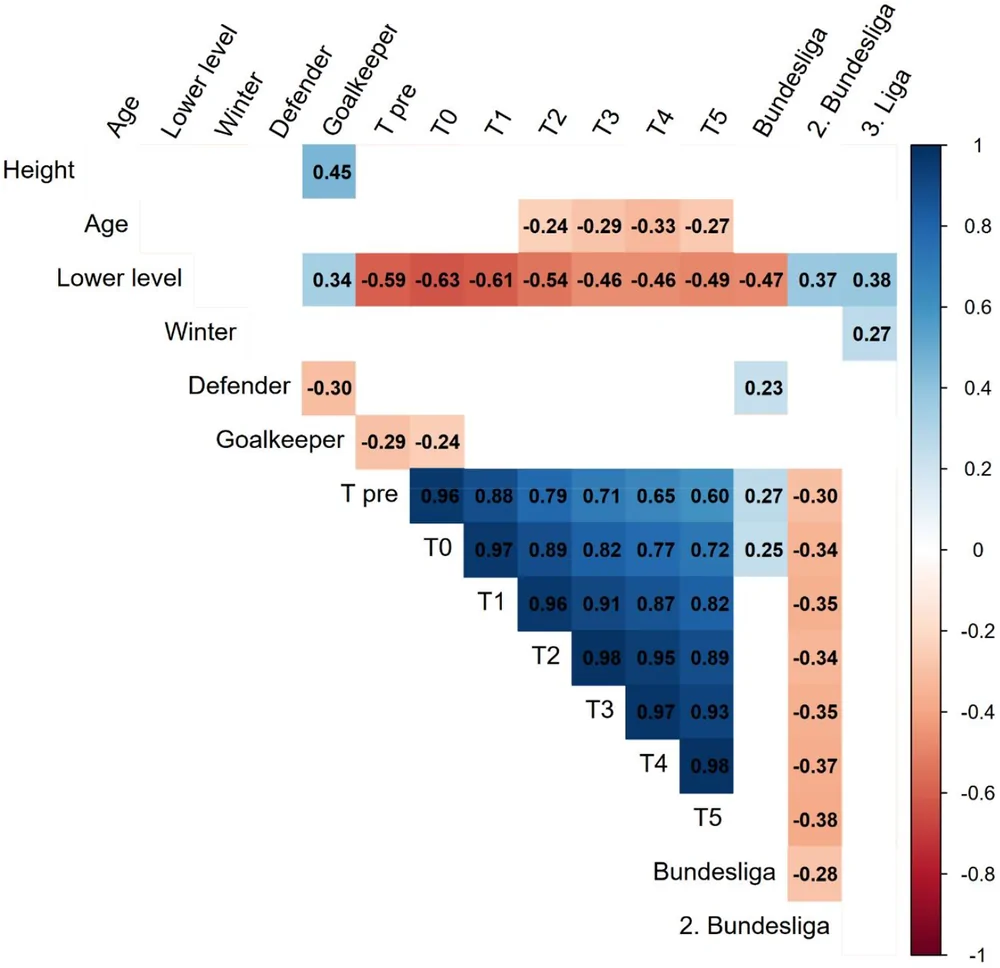
Significant Pearson correlations between player characteristics, competitive level and market value before and after meniscal injury.

Preinjury market value was strongly correlated with market value at the time of injury (*r* = 0.96) and during subsequent follow‐up, although the strength of this association progressively decreased from T1 (*r* = 0.88) to T5 (*r* = 0.60). Market values at consecutive postinjury assessments were also strongly correlated, with coefficients ranging from 0.96 to 0.98.

Age at injury was negatively correlated with market value at T2 (*r* = −0.24), T3 (*r* = −0.29), T4 (*r* = −0.33) and T5 (*r* = −0.27), indicating lower medium‐ and long‐term market values among older players.

Participation in lower‐tier competitions was negatively correlated with market value across all assessment points, including Tpre (*r* = −0.59), T0 (*r* = −0.63), T1 (*r* = −0.61), T2 (*r* = −0.54), T3 (*r* = −0.46), T4 (*r* = −0.46) and T5 (*r* = −0.49). Participation in the 2. Bundesliga was also negatively correlated with market value throughout the observation period, with coefficients ranging from −0.30 at Tpre to −0.38 at T5. Conversely, participation in the Bundesliga was positively correlated with preinjury market value (*r* = 0.27) and market value at T0 (*r* = 0.25).

Goalkeeper status was negatively correlated with market value at Tpre (*r* = −0.29) and T0 (*r* = −0.24) and positively correlated with player height (*r* = 0.45). No significant correlations were observed for injury year, duration of injury‐related absence, forward or midfielder position, or most individual leagues.

### Longitudinal changes in market value

Market value was assessed for 80 injury episodes before injury and during the injury year, with available observations decreasing to 79 at T1, 77 at T2, 73 at T3, 69 at T4, and 59 at T5. The mean market value increased from €4.58 million before injury to €5.13 million during the injury year and reached €5.46 million at T1. Thereafter, mean market value progressively declined to €5.18 million at T2, €4.60 million at T3, €4.17 million at T4 and €3.77 million at T5 (Table 2).

**Table 2:** Longitudinal changes in market value before and after meniscal injury in professional football players.

	*N*	Mean ± SD^a^ (K€)	Median [range] (K€)	% change versus Tpre (95% CI)^b^	*p*‐Value	% change versus T0 (95% CI)^b^	*p*‐Value
Tpre	80	4583.38 ± 8060.56	1000.00 [50.00, 40000.00]	Ref	–	−16.83 (−31.62; 1.15)	0.065
T0	80	5132.94 ± 8583.12	1200.00 [60.00, 45000.00]	20.24 (−1.13; 46.24)	0.065	Ref	–
T1	79	5456.58 ± 9036.61	1000.00 [45.00, 45000.00]	25.61 (3.18; 52.90)	0.023*	4.46 (−14.19; 27.16)	0.663
T2	77	5179.81 ± 8150.99	1500.00 [45.00, 40000.00]	26.82 (4.01; 54.64)	0.019*	5.47 (−13.50; 28.61)	0.598
T3	73	4596.99 ± 7683.83	1200.00 [30.00, 35000.00]	9.66 (−10.37; 34.17)	0.369	−8.80 (−25.46; 11.58)	0.370
T4	69	4165.14 ± 7515.40	1000.00 [20.00, 40000.00]	−13.37 (−29.44; 6.37)	0.170	−27.95 (−41.32; −11.54)	0.002*
T5	59	3774.49 ± 7803.02	1000.00 [20.00, 45000.00]	−31.56 (−44.84; −15.09)	0.001*	−43.08 (−54.12; −29.38)	<0.001*

Abbreviations: CI, confidence interval; Ref, reference category; SD, standard deviation.

^a^ Unadjusted mean and SD.

^b^ Estimated per cent change calculated using a mixed‐effects model to account for repeated measurements within each injury episode. Model ICC: 0.853; between‐episode variance: 2.30 (SD = 1.52); residual variance: 0.40 (SD = 0.63).

* *p* < 0.05.

When the preinjury season was used as the reference category, market value increased by 20.24% during the injury year, although this difference did not reach statistical significance (95% CI, −1.13% to 46.24%; *p* = 0.065). Significant increases were observed at T1 (+25.61%; 95% CI, 3.18% to 52.90%; *p* = 0.023) and T2 (+26.82%; 95% CI, 4.01% to 54.64%; *p* = 0.019). Market value subsequently approached preinjury levels at T3 (+9.66%; 95% CI, −10.37% to 34.17%; *p* = 0.369) and declined at T4 (−13.37%; 95% CI, −29.44% to 6.37%; *p* = 0.170), although neither difference was statistically significant. By T5, market value was significantly lower than the preinjury value (−31.56%; 95% CI, −44.84% to −15.09%; *p* = 0.001) (Figure 3a).

**Figure 3:**
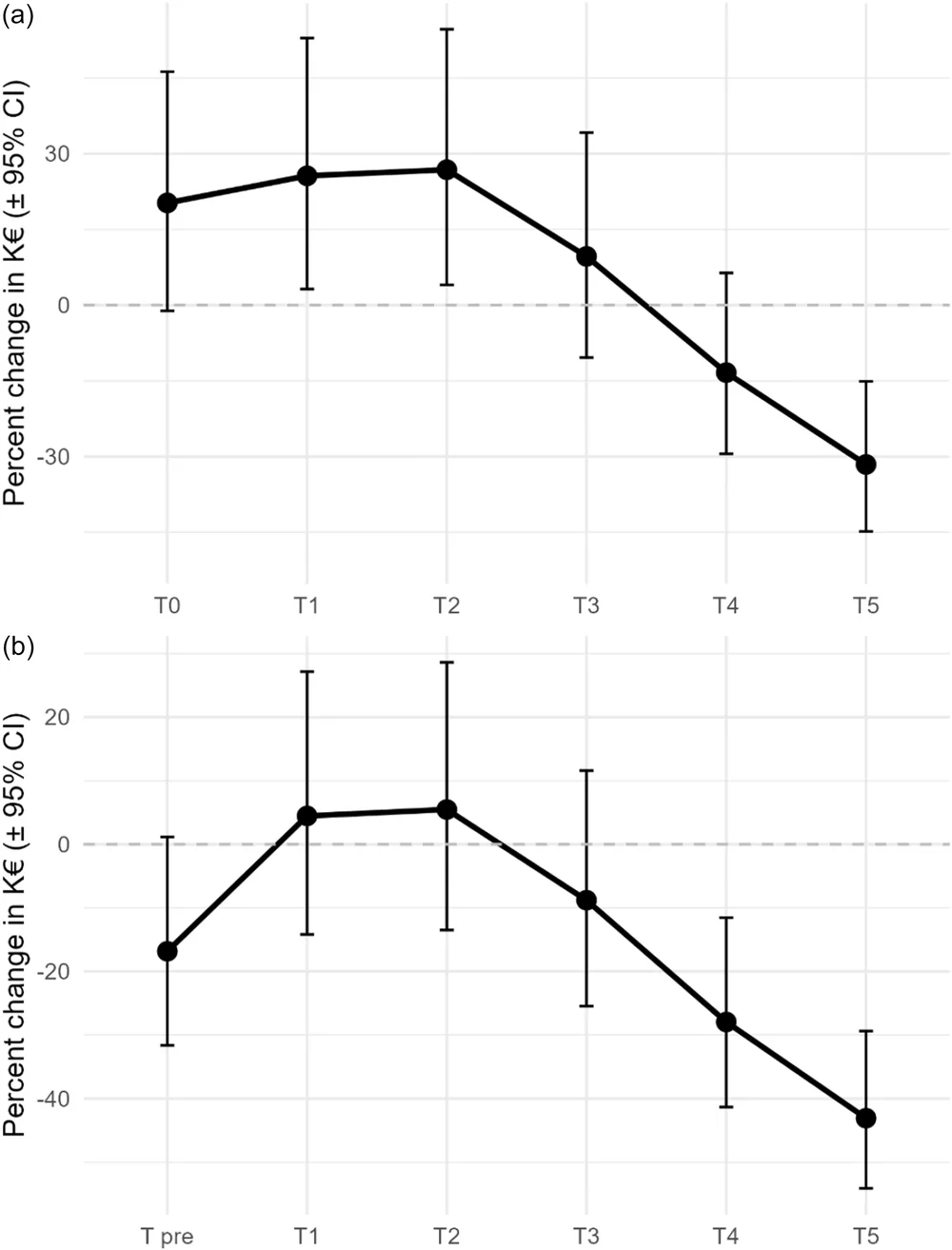
Estimated percentage changes in market value relative to the preinjury season (Panel a) and injury year (Panel b). The figure shows the estimated per cent change calculated using a mixed‐effects model to account for within‐subject repeated measurements.

When the injury year was used as the reference category, no significant differences were observed at T1 (+4.46%; 95% CI, −14.19% to 27.16%; *p* = 0.663), T2 (+5.47%; 95% CI, −13.50% to 28.61%; *p* = 0.598) or T3 (−8.80%; 95% CI, −25.46% to 11.58%; *p* = 0.370). Significant reductions emerged at T4 (−27.95%; 95% CI, −41.32% to −11.54%; *p* = 0.002) and became more pronounced at T5 (−43.08%; 95% CI, −54.12% to −29.38%; *p* < 0.001) (Figure 3b).

The mixed‐effects model yielded an ICC of 0.853, indicating that 85.3% of the total variance was attributable to differences between injury episodes.

When the analysis was restricted to the first episode of each player (*n* = 74), estimates were consistent with those of the full cohort (T1, +24.81%; *p* = 0.033; T2, +26.35%; *p* = 0.026; T5, −34.50%; *p* < 0.001). Trajectories did not differ significantly between tear‐specific and nonspecific injury labels at any time point (all interaction *p* ≥ 0.59) or between episodes with injury‐related absence above and below the median of 63 days (all interaction *p* ≥ 0.15); point estimates for longer absences were, if anything, more favourable (Supporting Information: Table S3).

### Comparison of market value by playing position

When market values across all assessment time points were considered, midfielders showed the highest mean market value (€5.86 ± €9.29 million), followed by forwards (€5.08 ± €7.84 million), defenders (€4.69 ± €8.07 million), and goalkeepers (€1.28 ± €2.08 million). However, with defenders used as the reference category, no statistically significant differences were observed for forwards (+12.72%; 95% CI, −60.26% to 219.72%; *p* = 0.820), goalkeepers (−62.14%; 95% CI, −88.37% to 23.24%; *p* = 0.105) or midfielders (+31.54%; 95% CI, −40.13% to 189.03%; *p* = 0.490) (Table 3).

**Table 3:** Overall comparison of market value according to playing position.

	*N*	Mean ± SD^a^ (K€)	Median [range] (K€)	% change (95% CI)^b^	*p*‐Value
Defender	36	4691.55 ± 8070.58	1000 [20, 45000]	Ref	–
Forward	11	5078.79 ± 7837.52	1250 [100, 35000]	12.72 (−60.26; 219.72)	0.820
Goalkeeper	8	1283.33 ± 2077.46	425 [50, 10000]	−62.14 (−88.37; 23.24)	0.105
Midfielder	25	5860.38 ± 9288.83	1500 [100, 45000]	31.54 (−40.13; 189.03)	0.490

Abbreviations: CI, confidence interval; Ref, reference category; SD, standard deviation.

^a^ Unadjusted mean and SD.

^b^ Estimated per cent change calculated using a mixed‐effects model to account for within‐subject repeated measurements. Model ICC: 0.836; between‐episode variance: 2.23 (SD = 1.49); residual variance: 0.44 (SD = 0.66).

Position‐specific comparisons at each time point similarly revealed no statistically significant differences in market value relative to defenders (Supporting Information: Table S1). Goalkeepers consistently had lower estimated market values than defenders, with differences ranging from −79.66% at Tpre to −40.67% at T5. The largest difference was observed during the preinjury season (−79.66%; 95% CI, −95.92% to 1.28%), although it did not reach statistical significance (*p* = 0.053). No significant differences were identified for forwards or midfielders at any assessment point.

### Multivariable analysis of market value trajectories

After adjustment for age at injury, playing position and competitive level, the preinjury annual change in market value was not statistically significant (+20.24%; 95% CI, −1.18% to 46.30%; *p* = 0.065). Entry into the postinjury period was associated with an upward level shift (+33.85%; 95% CI, 9.03% to 64.34%; *p* = 0.005), followed by a significant deterioration in the subsequent annual trajectory, as indicated by the time‐by‐postinjury interaction (−28.85%; 95% CI, −41.86% to −12.93%; *p* = 0.001) (Table 4).

**Table 4:** Multivariable mixed‐effects analysis of market value following meniscal injury.

	% change (95% CI)^a^	*p*‐Value
Time (per year)	20.24 (−1.18; 46.30)	0.065
Postinjury period (T > T0)	33.85 (9.03; 64.34)	0.005*
Age at injury (per year)	−9.45 (−15.22; −3.29)	0.004*
Role (defender as reference)		
Forward	14.44 (−51.24; 168.59)	0.754
Goalkeeper	−12.62 (−68.49; 142.36)	0.793
Midfielder	54.58 (−19.83; 198.04)	0.190
Competitive level (level 1 as reference)		
Level 2	−77.76 (−87.76; −59.59)	<0.001*
Level 3	−92.75 (−97.76; −76.51)	<0.001*
Time × postinjury interaction	−28.85 (−41.86; −12.93)	0.001*

Abbreviation: CI, confidence interval; SD, standard deviation.

^a^ Estimated per cent change calculated using a multiple mixed‐effects model to account for within‐subject repeated measurements. Model ICC: 0.784; between‐episode variance: 1.44 (SD = 1.20); residual variance: 0.40 (SD = 0.63).

* *p* < 0.05.

Age at injury was independently associated with lower market value, with a 9.45% reduction per additional year of age (95% CI, −15.22% to −3.29%; *p* = 0.004). Compared with players competing in first‐tier leagues, those in second‐ and third‐tier competitions had significantly lower market values (−77.76%; 95% CI, −87.76% to −59.59%; *p* < 0.001, and −92.75%; 95% CI, −97.76% to −76.51%; *p* < 0.001, respectively).

No significant differences were observed according to playing position, with defenders serving as the reference category. The ICC was 0.784, indicating that 78.4% of the total variance was attributable to differences between injury episodes. Model‐based predicted market value trajectories according to playing position and competitive level, calculated at the median age of 25 years, are presented in Figure 4.

**Figure 4:**
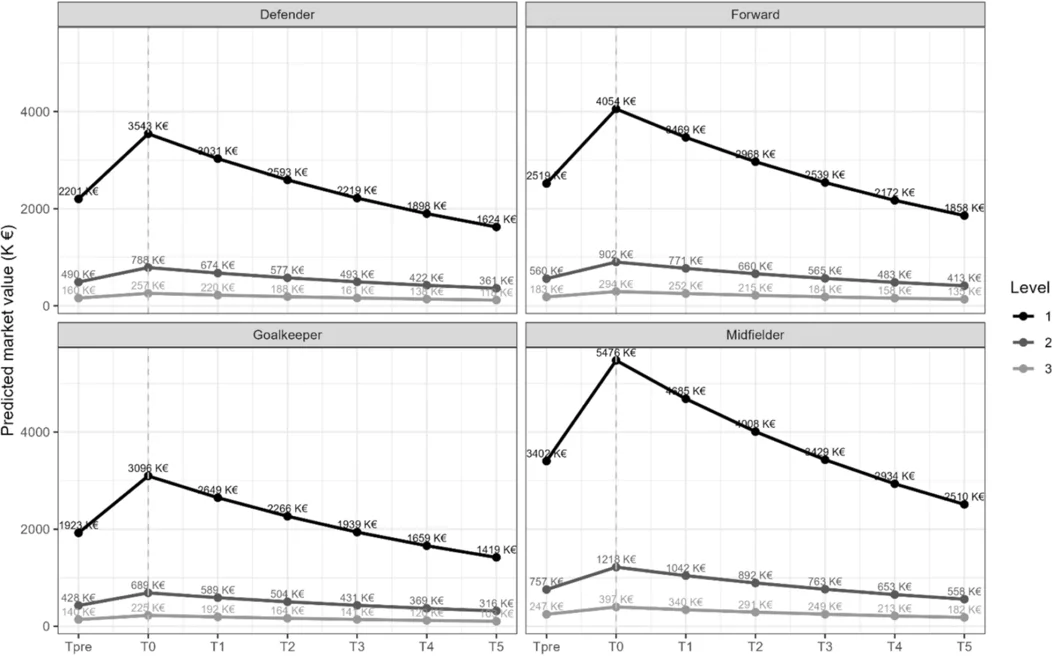
Predicted market value trajectories according to playing position and competitive level. Predictions are calculated at median age (25 years); the vertical dotted line represents the year of the injury.

### Comparison with matched controls

In this second stage, injured players were compared with matched controls (64 injured episodes and 128 controls), with market value measured identically for both groups. After matching, all SMDs were below 0.15 (age, 0.02; preinjury market value, 0.14; calendar year, 0.00; height, −0.03; playing position, 0.00; competitive level, 0.00; preferred foot, 0.13) (Table 5).

**Table 5:** Baseline characteristics of injured players and matched controls included in the matched analysis.

	Injured episodes (*n* = 64)	Matched controls (*n* = 128)	SMD
Age at injury/index date (years), mean ± SD	25.5 ± 4.1	25.4 ± 3.9	0.02
Preinjury market value (K€), median [IQR]^a^	1700 [662–4625]	1425 [500–4000]	0.14
Calendar year of injury/index date, mean ± SD	2015.5 ± 4.7	2015.5 ± 4.7	0.00
Height (cm), mean ± SD^b^	182.7 ± 5.5	182.9 ± 6.7	−0.03
Playing position, *n* (%)			0.00
Defender	35 (54.7)	70 (54.7)	
Midfielder	12 (18.8)	24 (18.8)	
Forward	13 (20.3)	26 (20.3)	
Goalkeeper	4 (6.2)	8 (6.2)	
Competitive level, *n* (%)			0.00
First tier	37 (57.8)	74 (57.8)	
Second tier	21 (32.8)	42 (32.8)	
Third tier or lower	6 (9.4)	12 (9.4)	
Preferred foot, *n* (%)^c^			0.13
Right	46 (71.9)	87 (68.0)	
Left	13 (20.3)	33 (25.8)	
Both	1 (1.6)	2 (1.6)	
Not available	4 (6.2)	6 (4.7)	

*Note*: Values for both groups were obtained from the same openly available, Transfermarkt‐derived dataset.

Abbreviations: IQR, interquartile range; SD, standard deviation; SMD, standardised mean difference.

^a^ SMD calculated on log‐transformed values; preinjury market value was a matching variable (caliper ± 50%).

^b^ Height missing for four injured players and five controls.

^c^ SMD calculated excluding missing values. For categorical variables, the multivariate SMD is reported.

Over the 5‐year period, market value declined in both groups. Relative to the preinjury season, injured players showed estimated changes of +15.8% at T0, −4.7% at T1, −7.0% at T2, −20.1% at T3, −24.9% at T4 and −44.8% at T5, whereas matched controls changed by +6.3%, +6.4%, +8.3%, +3.6%, −12.4% and −34.5%, respectively. In the mixed‐effects model, no between‐group difference reached statistical significance at any time point; the difference in change from baseline between injured players and controls was −15.7%; 95% CI, − 39.2% to +16.8%; *p* = 0.304 at 5 years, and the largest point difference was observed at T3 (−22.8%; 95% CI, −42.8% to +4.1%; *p* = 0.089) (Table 6, Figure 5). The long‐term reduction in market value among injured players was therefore not significantly greater than that of matched peers, although the point estimates from T1 onward favoured the controls.

**Table 6:** Longitudinal change in market value in injured players versus matched controls.

Time point	Injured (%)	Controls (%)	Difference (95% CI)	*p*‐Value
T0	+15.8	+6.3	+9.0 (−19; +46)	0.563
T1	−4.7	+6.4	−10.4 (−33; +20)	0.461
T2	−7.0	+8.3	−14.1 (−36; +15)	0.313
T3	−20.1	+3.6	−22.8 (−43; +4)	0.089
T4	−24.9	−12.4	−14.2 (−37; +17)	0.332
T5	−44.8	−34.5	−15.7 (−39; +17)	0.304

*Note*: values are estimated percentage changes from the preinjury season (Tpre) (64 injured episodes, 128 controls). The difference column reports the injured‐versus‐control contrast from the linear mixed‐effects group‐by‐time‐point interaction. In this matched analysis, market values for both groups were obtained from the same public source to ensure identical measurement; the primary analysis (Tables 1, 2, 3, 4) is unchanged. Available observations (injured/controls): Tpre 64/128, T0 64/128, T1 64/128, T2 61/119, T3 59/118, T4 52/109, T5 43/96.

Abbreviation: CI, confidence interval.

**Figure 5:**
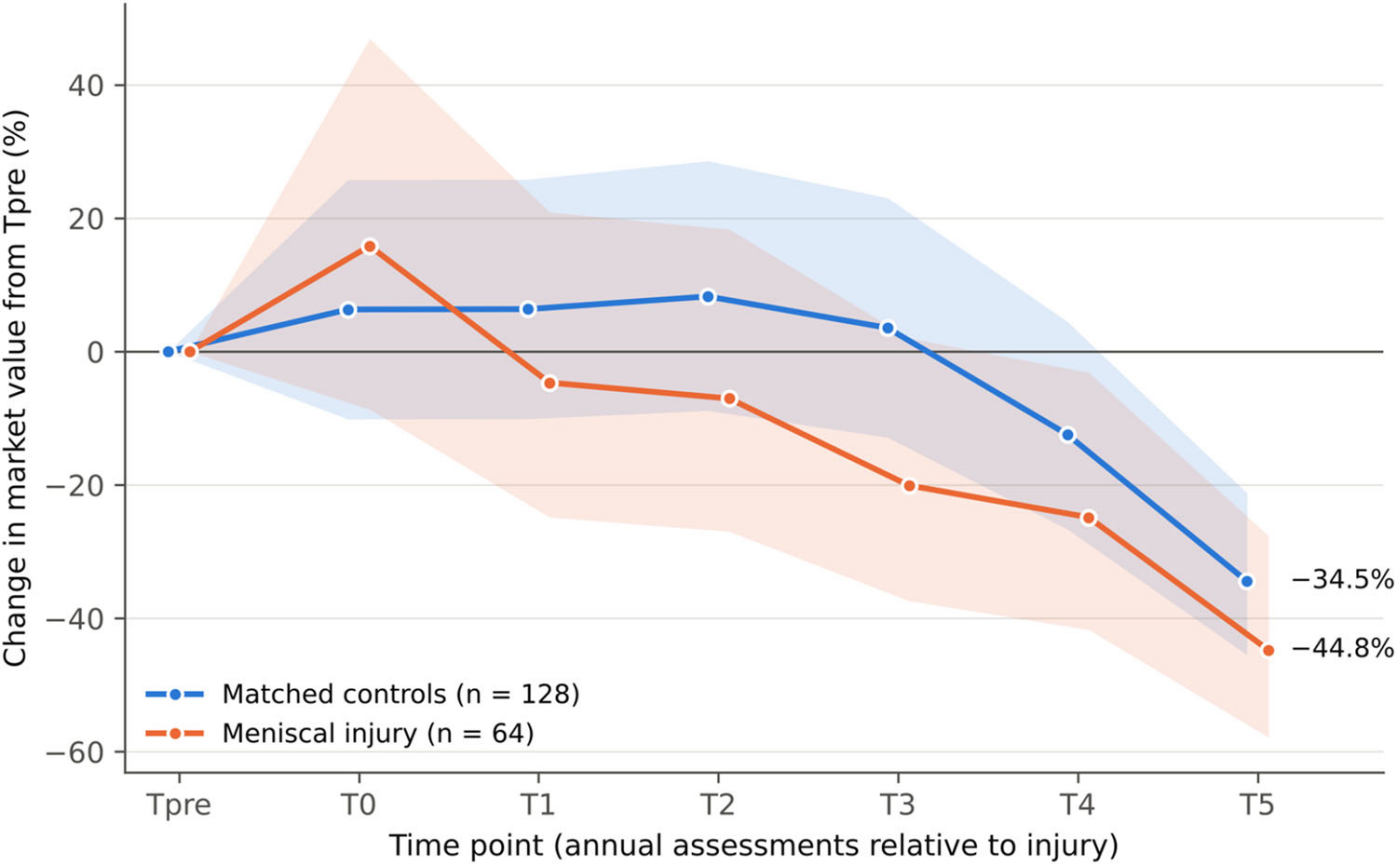
Estimated percentage change in market value from the preinjury season (Tpre) in injured players and matched controls. Shaded bands represent 95% confidence intervals. Estimates from the linear mixed‐effects group‐by‐time‐point model (64 injured episodes, 128 controls).

In sensitivity analyses, matching without competitive level yielded a 5‐year difference of +2.5% (95% CI, −25.4% to +40.8%; *p* = 0.878). When the control pool was restricted to players without any recorded lower‐extremity injury (108 controls), controls declined more than injured players (5‐year difference, +26.3%; 95% CI, − 8.5% to +74.4%; *p* = 0.156; T4, +50.3%; 95% CI, +10.7% to +104.1%; *p* = 0.009). Across 100 alternative random seeds, the median 5‐year difference was −7.4% (interquartile range, −14.7% to −1.0%), and a statistically significant 5‐year difference occurred in 2 of 100 draws (Supporting Information: Table S3).

## DISCUSSION

This study examined the longitudinal trajectory of market value after meniscal injury in professional football players and, for the first time, compared injured players with matched controls. In the primary analysis of 80 injury episodes, market value did not fall immediately after injury; it rose over the first two seasons and then declined, reaching values significantly below the preinjury season by 5 years. In the matched analysis, however, this long‐term decline was also observed in peers matched for age, position, competitive level, value and calendar year, with no statistically significant between‐group difference at any time point. Taken together, these findings indicate that meniscal injury is not associated with an immediate loss of market value and that the later decline largely mirrors the expected age‐related trajectory of player value. Because the point estimates favoured the controls from T1 onward and the confidence intervals were wide, the results are best interpreted as showing no clear additional decline in market value within this cohort, rather than as excluding an injury‐related effect.

The absence of an immediate reduction is consistent with the way market value is generated: it integrates expected future performance and is relatively inert to a single, well‐managed injury from which a player is expected to recover [14]. The progressive divergence from the preinjury season over subsequent years—the pattern captured in the notion of a cost ‘beyond return to play’—initially appears to suggest a delayed economic cost. The controlled analysis reframes this observation: because matched peers of the same age and value experienced a substantial decline, the late reduction is better understood largely as the normal downslope of the age–value curve, which for players injured in their mid‐twenties begins within the follow‐up window, rather than primarily as a lasting consequence of the meniscal injury itself.

A central methodological question in single‐arm longitudinal designs is whether an observed change reflects the exposure or the natural history of the outcome. Player market value depends strongly and nonlinearly on age, peaking in the mid‐to‐late twenties and declining thereafter; a cohort injured at a mean age of 25 years is therefore expected to lose value over a 5‐year horizon irrespective of injury. To separate the effect of meniscal injury from this background trajectory, injured players were compared with a matched control group, following the approach used by Lavoie‐Gagne and colleagues, who matched uninjured controls to elite soccer players with meniscal tears [14]. Controls were required to have no recorded meniscal injury and were matched on playing position, competitive level, age, preinjury market value and calendar period, so that the two groups differed principally in the occurrence of the meniscal injury, while market value was measured using an identical method in both groups; balance after matching was confirmed for all measured covariates.

This design converts the question from whether value fell after injury—to which the answer is trivially affirmative, as it would be for any aging player—into the more informative question of whether value fell more than in comparable players, for which no statistically significant difference was found. The estimates, however, were imprecise: the 5‐year contrast was compatible with an injury‐related difference ranging from a moderate loss to a modest gain, and its direction varied across matching specifications. A moderate injury‐related effect, therefore, cannot be excluded, and larger cohorts would be required to detect differences of this magnitude.

The rise in market value observed during the first two seasons in the primary analysis should be interpreted cautiously. It is consistent with the continued maturation of players who returned to play and were, on average, still approaching their peak value, and it may partly reflect survivorship, since players who did not return or who retired contributed fewer later observations. The matched comparison is again informative: when market value was re‐measured with an identical anniversary‐based definition in both groups, the early rise was limited to the injury year, after which injured players remained below controls without a statistically significant difference. The early increase in the primary analysis is therefore partly attributable to the season‐based definition of market value and should not be interpreted as an injury‐related gain.

Among the factors examined in the primary multivariable analysis, older age and lower competitive level were independently associated with lower market value, whereas playing position was not. These associations are expected, as value declines with age and is strongly stratified by the tier of competition. The absence of a positional effect, despite goalkeepers showing numerically lower values, is likely explained by limited power within positional subgroups. Because competitive level and age—the dominant determinants of value—were explicitly balanced in the matched analysis, the interpretation that the meniscal injury contributed little independent long‐term effect is further supported.

These results contrast with the growing literature on anterior cruciate ligament injuries in professional football, where injury has been linked to measurable reductions in performance, career length, and economic value [5, 6, 12], and with the significant decline in market value reported after proximal hamstring repair in elite players [4]. The divergence is clinically coherent: anterior cruciate ligament rupture is a more severe injury with a longer and less certain recovery, whereas meniscal injury—although associated with prolonged absence [8, 16] and, after meniscectomy, an increased long‐term risk of osteoarthritis [13, 17]—carries a comparatively favourable short‐to‐medium‐term prognosis, with high rates of return to play [2, 14]. For clubs, agents and medical staff, the practical implication is cautiously reassuring: within this cohort, a meniscal injury was not followed by a clear additional decline in market value beyond the expected age‐related trajectory. Given the sample size and the lack of information on injury severity, associated pathology, and treatment, these findings do not exclude an injury‐related effect in individual players, and contract and transfer decisions should continue to be based on individual clinical assessment.

### Clinical relevance

From a clinical perspective, the decision between meniscal repair and meniscectomy in elite players is frequently influenced by pressure for a rapid return, since return to sport takes approximately 2 months after meniscectomy and approximately 6 months after repair [2], with lesion‐specific rehabilitation after repair [3]. Meniscal preservation, however, remains essential for long‐term joint health [13, 17], and repair of selected tear patterns achieves high rates of return to sport [7]. In the present cohort, a longer documented absence—which partly reflects more complex lesions and repair procedures—was not associated with a less favourable market value trajectory. Although treatment‐specific data were not available, these findings suggest that the anticipated economic consequences of a longer absence should not, by themselves, discourage meniscal preservation when it is clinically indicated. The results also provide team physicians with objective information for counselling players and clubs and illustrate that matched designs are required to distinguish injury‐related changes from the natural evolution of players' careers when publicly available sports data are used [11].

The principal strengths of this study are its longitudinal design over an 18‐year period, the use of mixed‐effects models accounting for repeated measurements, and, above all, the addition of a matched control group in which market value was measured identically in both arms, balance after matching was verified and robustness was assessed across several matching specifications and random seeds—features that strengthen causal interpretation relative to single‐arm designs.

### Limitations

This study has several limitations. First, market value is a crowd‐sourced estimate rather than an actual transfer fee; although widely used as a proxy for players' economic and performance status, it may not capture every financial consequence of injury. Second, the matched analysis was necessarily restricted to the 64 of 80 injury episodes for which a complete market‐value trajectory could be reconstructed in the openly available database; of the 16 excluded episodes, 10 involved players who were not indexed in that database and 6 involved players present but lacking baseline valuations. These episodes were disproportionately, though not exclusively, players competing in lower divisions (second tier and below) or with older or less completely recorded careers, while a minority were top‐division players absent from the database. The matched comparison should therefore be interpreted as applying primarily to players competing in the second division and above, and residual selection related to database coverage cannot be excluded. In addition, market values in the matched analysis were defined on anniversary dates and therefore differ in absolute magnitude from the season‐based values of the primary analysis; the between‐group contrasts, rather than the absolute changes, should be compared across analyses. Third, control players had no recorded meniscal injury in the available database; however, unreported or incompletely recorded injuries cannot be excluded. Moreover, controls may have sustained other injuries. Any misclassification of injury status could have attenuated between‐group differences; conversely, restricting controls to players without any recorded lower‐extremity injury selected players with fewer recorded events and steeper value decline, which illustrates the sensitivity of the comparison to control definition. Fourth, matching was performed on competitive level rather than on exact league, and detailed injury information—tear morphology, affected compartment (medial or lateral), concomitant injuries and treatment (meniscectomy or repair)—was not available. Transfermarkt injury categories specified the lateral meniscus in only 5 of 80 episodes and never specified the medial meniscus or the treatment; the analyses based on injury labels and on injury‐related absence are therefore only indirect surrogates and do not replace clinically verified data. Because treatment influences time to return and long‐term joint health [2, 13, 17], future studies linking clinical records or imaging to economic outcomes are warranted. Fifth, the primary cohort was derived from a previously compiled extraction that was not designed as a complete census of all meniscal injuries recorded on Transfermarkt; it therefore represents a sample rather than the full population of such injuries, which limits the precision of the estimates and their generalisability. A larger study based on all eligible meniscal injuries in publicly available datasets is planned to confirm these findings. Finally, the observational design precludes causal inference, statistical power for subgroup and interaction analyses was limited, and injury‐related absence does not equate to confirmed return to match play. These findings should therefore be regarded as hypothesis‐generating and warrant confirmation in larger, clinically detailed cohorts.

## CONCLUSIONS

Meniscal injuries were not associated with an immediate reduction in market value among professional football players. The long‐term decline in market value did not differ significantly from that of matched players without meniscal injury and appeared largely to reflect the expected age‐related evolution of player value; however, given the width of the confidence intervals and the absence of clinical detail, a moderate injury‐related effect cannot be excluded.

## AUTHOR CONTRIBUTIONS

**Riccardo D'Ambrosi**: Conception; design; writer; materials; data collection; literature review. **Jacopo Conteduca**: Design; data collection; processing; writer, materials, literature review. **Andrea Mei**: Writer; critical review; literature review; interpretation. **Marco Sorrentino**: Writer; critical review; literature review; processing; interpretation. **Claudio Cordani**: Conception; supervision; interpretation; critical review. **Christian Fink**: writer; critical review; literature review; processing. **Philipp Baumert**: Conception; supervision; interpretation; critical review.

## CONFLICT OF INTEREST STATEMENT

The authors declare no conflicts of interest.

## ETHICS STATEMENT

The authors have nothing to report.

## Supporting information

Supporting File 1

## Data Availability

Available upon reasonable request to the corresponding author.
