# Supplementary material for: Long‐term market value after meniscal injury in professional football players largely follows expected career trajectories: A matched cohort study
Source: J Exp Orthop. 2026 Oct 29;13(4):e70968. doi: 10.1002/jeo2.70968 (PMC13718456; doi:10.1002/jeo2.70968)
Supplement: Supplementary file 1 [file JEO2-13-e70968-s001.docx]

**Supplementary Material**

*Long-Term Market Value After Meniscal Injury in Professional Football Players Largely Follows Expected Career Trajectories: A Matched Cohort Study*

**Supplementary Table S1. Comparison of Market Value According to Playing Position at Each Assessment Time Point (formerly Table 4)**

| **Time point** | **Position** | **N** | **Mean ± SD ¹ (K€)** | **Median [range] (K€)** | **% change (95% CI) ²** | **p** |
| --- | --- | --- | --- | --- | --- | --- |
| Tpre | Defender | 36 | 5259.58 ± 9313.02 | 1000.00 [70.00, 40000.00] | Ref | – |
|  | Forward | 11 | 3522.73 ± 4304.84 | 1000.00 [100.00, 14000.00] | -2.70 (-76.37; 300.58) | >0.99 |
|  | Goalkeeper | 8 | 515.62 ± 483.47 | 375.00 [50.00, 1200.00] | -79.66 (-95.92; 1.28) | 0.053 |
|  | Midfielder | 25 | 5378.00 ± 8498.20 | 2500.00 [150.00, 38000.00] | 38.22 (-52.56; 302.73) | 0.834 |
| T0 | Defender | 36 | 5783.61 ± 9388.65 | 1350.00 [60.00, 45000.00] | Ref | – |
|  | Forward | 11 | 4168.18 ± 5194.67 | 1000.00 [100.00, 15000.00] | -11.99 (-78.19; 255.17) | 0.994 |
|  | Goalkeeper | 8 | 1178.12 ± 1983.66 | 625.00 [75.00, 6000.00] | -73.12 (-94.48; 30.88) | 0.130 |
|  | Midfielder | 25 | 5886.00 ± 9730.99 | 2000.00 [200.00, 38000.00] | 18.04 (-58.87; 238.77) | 0.971 |
| T1 | Defender | 36 | 5445.00 ± 8337.76 | 1000.00 [45.00, 40000.00] | Ref | – |
|  | Forward | 11 | 5022.73 ± 7304.12 | 3000.00 [150.00, 25000.00] | 1.04 (-74.84; 305.75) | >0.99 |
|  | Goalkeeper | 8 | 1793.75 ± 3372.52 | 600.00 [100.00, 10000.00] | -65.28 (-92.83; 68.11) | 0.276 |
|  | Midfielder | 24 | 6893.75 ± 11729.33 | 1750.00 [250.00, 45000.00] | 27.62 (-55.94; 269.63) | 0.918 |
| T2 | Defender | 36 | 4317.92 ± 6122.19 | 900.00 [45.00, 25000.00] | Ref | – |
|  | Forward | 10 | 6980.00 ± 10943.34 | 2000.00 [250.00, 35000.00] | 48.50 (-62.98; 495.65) | 0.857 |
|  | Goalkeeper | 8 | 1737.50 ± 2714.08 | 400.00 [150.00, 8000.00] | -54.26 (-89.98; 108.91) | 0.497 |
|  | Midfielder | 23 | 6943.48 ± 10406.79 | 1800.00 [200.00, 40000.00] | 61.89 (-42.62; 356.79) | 0.580 |
| T3 | Defender | 33 | 3968.33 ± 6651.75 | 1200.00 [30.00, 35000.00] | Ref | – |
|  | Forward | 9 | 6069.44 ± 10302.53 | 1500.00 [325.00, 32000.00] | 37.14 (-67.44; 477.67) | 0.927 |
|  | Goalkeeper | 8 | 1475.00 ± 2042.41 | 325.00 [200.00, 6000.00] | -53.48 (-89.69; 109.93) | 0.507 |
|  | Midfielder | 23 | 6008.70 ± 9046.35 | 1500.00 [150.00, 35000.00] | 39.39 (-50.67; 293.83) | 0.809 |
| T4 | Defender | 32 | 4092.81 ± 7864.91 | 1500.00 [20.00, 40000.00] | Ref | – |
|  | Forward | 7 | 5825.00 ± 10072.50 | 1000.00 [100.00, 28000.00] | 14.37 (-79.04; 524.06) | 0.996 |
|  | Goalkeeper | 8 | 1156.25 ± 1654.09 | 325.00 [100.00, 5000.00] | -54.93 (-90.97; 124.90) | 0.527 |
|  | Midfielder | 22 | 4836.36 ± 7538.76 | 1100.00 [100.00, 28000.00] | 22.81 (-60.18; 278.74) | 0.956 |
| T5 | Defender | 29 | 3642.93 ± 8654.08 | 1000.00 [20.00, 45000.00] | Ref | – |
|  | Forward | 7 | 4307.14 ± 7953.64 | 500.00 [100.00, 22000.00] | 3.20 (-80.57; 448.10) | >0.99 |
|  | Goalkeeper | 6 | 1075.00 ± 1469.95 | 525.00 [150.00, 4000.00] | -40.67 (-89.98; 251.26) | 0.844 |
|  | Midfielder | 17 | 4732.35 ± 7835.32 | 1000.00 [150.00, 30000.00] | 60.90 (-52.08; 440.20) | 0.696 |

¹ Unadjusted mean and SD; ² estimated percent change. Abbreviations: CI = confidence interval; Ref = reference category; SD = standard deviation.

**Supplementary Table S2. Transfermarkt Injury Categories Used to Define Meniscal Injury**

| **Code** | **Original label (German)** | **English translation** | **Episodes, n (%)** | **Injury-related absence (days), median [IQR]** |
| --- | --- | --- | --- | --- |
| 211 | Meniskusverletzung | Meniscal injury | 31 (38.8) | 56 [32–85] |
| 7 | Meniskusschaden | Meniscal damage | 19 (23.8) | 98 [56–180] |
| 150 | Meniskuseinriss | Meniscal tear | 14 (17.5) | 60 [48–94] |
| 348 | Meniskusriss | Meniscal rupture | 11 (13.8) | 131 [72–224] |
| 151 | Außenmeniskuseinriss | Lateral meniscal tear | 5 (6.2) | 96 [66–264] |
| Total |  |  | 80 (100) | 63 [43–137] |

Codes and labels correspond to the injury classification recorded in the Transfermarkt extraction. No category specified the medial meniscus, tear morphology, concomitant injuries, or the treatment performed. In the openly available dataset used for the matched analysis, the corresponding English labels were “Meniscus injury”, “Meniscus damage”, “Meniscus tear”, “Tear of the lateral meniscus”, and “Meniscus irritation”. One absence value was missing. IQR = interquartile range.

**Supplementary Table S3. Sensitivity Analyses**

| **Analysis** | **Estimate 1** | **Estimate 2** | **Estimate 3** | **Interpretation** |
| --- | --- | --- | --- | --- |
| A. Primary longitudinal cohort (single-arm) |  |  |  |  |
| First episode per player (n = 74), change vs Tpre | T1: +24.81 (+1.76; +53.08); p = 0.033 | T2: +26.35 (+2.83; +55.27); p = 0.026 | T5: −34.50 (−47.68; −18.00); p < 0.001 | Consistent with full cohort |
| Tear-specific (n = 30) vs nonspecific labels (n = 50), interaction | T1: −10.5 (−41; +35); p = 0.595 | T3: −1.5 (−35; +50); p = 0.945 | T5: +1.7 (−35; +59); p = 0.942 | No difference at any time point (all p ≥ 0.59) |
| Absence > 63 days (n = 39) vs ≤ 63 days (n = 40), interaction | T1: +20.3 (−19; +79); p = 0.361 | T3: +34.7 (−10; +102); p = 0.149 | T5: +28.7 (−17; +99); p = 0.255 | No difference (all p ≥ 0.15) |
| B. Matched analysis (difference injured − controls) |  |  |  |  |
| Main specification (64 vs 128; competitive level matched) | T1: −10.4 (−33.0 to +19.9); p = 0.461 | T3: −22.8 (−42.8 to +4.1); p = 0.089 | T5: −15.7 (−39.2 to +16.8); p = 0.304 | No significant difference |
| Without matching on competitive level (64 vs 128) | T1: +5.7 (−20.2 to +39.9); p = 0.699 | T3: +1.2 (−24.2 to +35.0); p = 0.938 | T5: +2.5 (−25.4 to +40.8); p = 0.878 | No significant difference |
| Controls without any recorded lower-extremity injury (64 vs 108) | T1: +20.6 (−9.3 to +60.3); p = 0.197 | T4: +50.3 (+10.7 to +104.1); p = 0.009 | T5: +26.3 (−8.5 to +74.4); p = 0.156 | Controls declined more; T4 significant in favor of injured players |
| 100 alternative random seeds (main specification), median [IQR] of differences | T1: −5.8 [−9.4; −0.6] | T3: −10.0 [−14.7; −3.2] | T5: −7.4 [−14.7; −1.0] | p < 0.05 at T5 in 2/100 draws |

Values are percentage changes or differences in percentage change with 95% confidence intervals, estimated with linear mixed-effects models (log market value) with a subject-specific random intercept. Interaction terms compare the change from Tpre between subgroups. In the analysis restricted to controls without any recorded lower-extremity injury, 8 injured episodes had no eligible control and 4 had one control. IQR = interquartile range.
